# Editorial for Special Issue: ZnO Nanostructures for Tissue Regeneration, Drug-Delivery and Theranostics Applications

**DOI:** 10.3390/nano11020296

**Published:** 2021-01-24

**Authors:** Valentina Cauda, Marco Laurenti

**Affiliations:** Department of Applied Science & Technology, Politecnico di Torino, Corso Duca degli Abruzzi 24, 10129 Torino, Italy

In recent years, zinc oxide (ZnO)-based nanomaterials have attracted a great deal of interest thanks to their outstanding and multifunctional properties. Actually, ZnO can be synthesized in a broad variety of nano-sized morphologies (such as nanowires, nanorods, nanoparticles, and nanoflowers), shows easy preparation routes and facile surface chemical functionalization. Most importantly, ZnO has many intriguing properties, being a semiconductor, piezoelectric, pyroelectric and photoexcitable material, with low chemical stability in acidic environments and interesting antimicrobial and anticancer properties. These aspects fostered a deep investigation of ZnO nanomaterials to design and fabricate smart biocompatible nanotools, which have been successfully applied to a wide plethora of applications in the biomedical field. In such cases, ZnO nanostructures, alone or combined into hybrid or composite systems, represent a powerful tool for the fabrication of new scaffolds for tissue regeneration with improved antimicrobial properties, as well as for drug delivery applications. Moreover, the promising optical and biocompatible properties of ZnO have been successfully combined together, resulting in the co-presence of imaging and therapeutic actions, useful for theranostics applications towards cancer therapy.

This Special Issue of *Nanomaterials* is therefore dedicated to the most recent advances in the use of ZnO nanostructures for designing novel smart nanomaterials dedicated to biomedical systems, tissue engineering, drug delivery and theranostics devices. It ranges from the synthesis and characterization of the starting nanomaterials, to their final in vitro applications.

To have a proper overview in the specific field of ZnO nanostructures for drug delivery and theranostics applications, the Review from Prof. Maria Vallet-Regì and coworkers [1] is very relevant. Here, the authors analyze recent strategies in proposing ZnO as semiconductor quantum dots (QDs) not only for bio-imaging purposes but also as multifunctional tools, i.e., for drug delivery and theranostic imaging against different diseases. In particular, the application of ZnO for antibacterial or anti-inflammatory treatments, against diabetes and cancer, as well as in wound healing are proposed with various in vitro and in vivo examples from the literature.

In the paper of Dr. Nadia Garino et al. [2], a special focus is given to the synthetic protocols applied to produce ZnO nanocrystals and their surface decoration by aminopropyl groups facing colloidal dispersion and stability over time when used towards cancer cells. Actually, the paper shows a novel microwave-assisted sol–gel synthetic route, pointing out how important it is to control all the nanomaterial properties when dealing with biological entities, i.e., living cells, for the achievement of reproducible and reliable results.

A similar relationship between the nanomaterials’ properties, the synthetic route and the interaction with the biological world (here microorganisms) is reported in the work of Prof.s Roberta Cavalli, Barbara Onida and coworkers [3]. Therein, wet organic-solvent-free processes were used to produce two ZnO nanostructures with different morphologies, yet providing different surface areas, crystal sizes, and thus dissolution rates into zinc cations. The antimicrobial effects of these ZnO nanostructures were then measured on various bacterial strains and the successful loading of the anti-inflammatory drug ibuprofen was successfully proposed for the first time using a supercritical CO_2_-mediated impregnation process. This paper demonstrates the potential use of ZnO nanomaterials as a multifunctional antimicrobial drug nanocarrier.

Concerning bone tissue engineering applications, the work of Prof. Maria Vallet-Regì and Antonio Salinas [4] shows that ZnO can be efficiently used in Mesoporous Bioactive Glasses (MBGs) as carriers for the peptide osteostatin. Interestingly, the zinc cations release from the MBG, combined with the osteogenic properties of osteostatin, provided a valuable tissue engineering device, as proved by in vitro tests with pre-osteoblasts.

Another representative in the field of tissue engineering is the work of M. Cierech et al. [5]; in this study, ZnO nanoparticles incorporated into a polymeric matrix were successfully designed to simultaneously show anti-bacterial effects and retention of both mechanical and hydrophilic properties useful for preparing a denture base.

As a concluding remark, with this Special Issue we hope we have contributed to highlight the role of zinc oxide nanomaterials in cancer cell theranostics, drug delivery and tissue engineering, providing insights from their synthesis, surface functionalization and characterization to their smart behaviors with customizable properties for advanced and personalized biomedical applications.
